# Life skills profile of patients with schizophrenia and its correlation to a feeling of rejection among key family carers

**DOI:** 10.4103/0019-5545.55953

**Published:** 2005

**Authors:** L.S.S Manickam, R. Satheesh Chandran

**Affiliations:** * Professor and Head, Department of Clinical Psychology, Sri Ramachandra Medical College and Research Institute (Deemed University), Chennai 600116; ** Assistant Director, Social Organization for Mental Health Action (SOMA) P.O. Box 6116, Thiruvananthapuram 695041

**Keywords:** Life skills profile, Patient Rejection Scale, family carers of patients with chronic schizophrenia

## Abstract

**Background:**

The behaviour of patients with schizophrenia is of great concern to key family carers. Life skills profile (LSP) is the measure that has considerable importance in assessing the functioning of people with schizophrenia in the community.

**Aim:**

To assess the sociodemographic correlates of LSP, and to find the correlation between LSP and the rejection response of key family carers.

**Methods:**

The LSP of 48 patients with chronic schizophrenia (29 men and 19 women) was assessed. The rejection responses of key family carers (28 men and 20 women) were evaluated using the Patient Rejection Scale.

**Results:**

The LSP did not significantly differ on the variables of gender, income level or attendance of day-care centre. However, there were differences between patients from urban and rural areas. A new dimension of family harmony, added as a subscale to LSP, also did not show any significant difference on the above variables. The rejection responses of key family carers were found to be significantly related to the LSP of the patients and, among the subscales, family harmony and communication were positively related to rejection.

**Conclusion:**

Though family interventions have been found to have positive implications on relapse and social functioning of patients with schizophrenia, a model of a family intervention programme for families of patients with schizophrenia needs to be developed.

## INTRODUCTION

The behaviour of patients with schizophrenia is of much concern to key family carers and other caregivers. Key family carers are disturbed by the deviant and disruptive behaviour of the patients, which manifests as social disability components.^1^ Though the diagnostic label of schizophrenia and its ‘stigma” bothers some key family carers who hail from the high socioeconomic strata, the majority of them are worried about the positive and negative symptoms and the long-term care required for such patients.^2^ Research shows that key family carers in India have a significant burden,^3^ are distressed,^4^ and receive little social support.^5^

Rao and Nagalakshmi studied the social competence of patients with schizophrenia. No statistically significant difference was found between the study group and control groups of neurotic depressives and normal individuals.^6^ However, a qualitative analysis showed that those with schizophrenia were non-assertive, unable to make and keep friends, and take decisions. The group also lacked tact, poise and spontaneity in narrating personal experiences.

The life skills profile (LSP) of people with schizophrenia was initially studied by Rosen *et al*.^7^ and they developed a measure for assessing the function and disability of patients with schizophrenia. The measure focuses on different aspects of the functions that affect survival and adaptation in the community. The five subscales of LSP are self-care, non-turbulence, social contact, communication and responsibility. Though different scales were developed to assess disability, adjustment, rehabilitation needs and performance-based skills,^8^ LSP appears to be of considerable use in assessing the functioning of patients with schizophrenia in the community.^9^

There has been an increased interest in the LSP of persons with schizophrenia and its various sociodemographic correlates.^9^–^11^ Norman *et al*.^12^ assessed community functioning and its association with neurocognitive measures using LSP, but did not find any significant correlation. LSP was found to predict community functioning rather than neurocognitive measures. On the other hand, Parker *et al*.^9^ observed that LSP measured the disability of people with schizophrenia, and did not predict outcome in community mental health to the full extent. In another attempt, Chopra *et al*.^13^ used LSP along with the WHO Disability Assessment Schedule II (WHO-DAS-II) and Health of the Nation Outcome Scales (HoNOS) to study the disability of 20 patients in the community and found HoNOS to be a better measure than the others. They attributed the superiority of the tool to its cultural sensitivity. Eagar *et al*.^14^ evaluated the outcome measures in public adult mental health services in New Zealand using LSP and found the tool suitable for measuring routine outcome.

Patients with schizophrenia living with their families often disrupt the daily routine of other members.^15^ Apart from the subjective distress and financial loss, there is also a risk to the safety of key family carers. Therefore, assessing the patient's skill in maintaining family harmony assumes importance. The skill of the patient with schizophrenia in maintaining non-disruptive interpersonal relationships, refraining from problem behaviours that disturb the functioning of the family unit as a whole and those which may weaken intergenerational (parent–child) or interpersonal (marital alliance) boundaries^16^ help maintain family harmony. Hence, in our sociocultural context, the component of family harmony should also be included to make LSP comprehensive.

The emotional reactions of key family carers are varied. Extensive research conducted so far has identified factors such as expressed emotion (EE),^17^ feeling of rejection,^18^ distress^4^ and burden.^5^ The perceptions of key family carers are assessed through different scales. The index of EE gives a combined score of critical comments, hostility and emotional over-involvement of key family carers and is obtained from the Camberwell family interview;^17^ it requires special training for administration. Kreisman *et al*.^18^ developed an 11-item Patient Rejection Scale (PRS) that assesses self-reported feelings of rejection; it is easy to administer.

An attitude of rejection among key family carers is associated with the symptoms and number of re-hospitalizations.^19^ Establishing a relationship between life-skills functioning of the patient with schizophrenia and the attitude of rejection of key family carers is likely to help plan a psychoeducational programme for key family carers and help in rehabilitation of the patient. An association of the feeling of rejection with any of the subscales of LSP may also help plan social skills' training for patients.

The objectives of the present study were (i) to assess the sociodemographic correlates of LSP, and (ii) find the correlation, if any, between LSP and the rejection response of key family carers.

## METHODS

### Sample

Subjects were chosen by purposive sampling. Key family members who were primarily responsible for giving care to the 48 patients (29 men and 19 women) with chronic schizophrenia were included in the study. The Consultant Psychiatrist at the Mental Health Centre, Thiruvananthapuram, diagnosed the patients according to the ICD-10 guidelines. The duration of illness of all the patients was more than 5 years and all were under medication. Twenty-eight patients were attending the day-care centre. Others were at home through the day either with their key family carers or other family members.

The subjects were contacted when they attended the outpatient department of the centre along with the patient for follow up or when they came to attend group meetings at the day-care centre. The composition of the key family carers of the patients (28 men and 20 women) was as follows: father 17, mother 15, husband 2, wife 2, brother 9, sister 1 and cousin 2. Their ages ranged from 17 to 57 years (mean 32.27 years). Of these, 29 were from urban areas and 19 from rural areas. Thirty-eight subjects belonged to the low-income group, 9 to the middle and 1 to the high-income group.

### Tools

#### Life Skills Profile

This is a 39-item scale with five subscales. Each subscale has varying number of items: self-care (10), non-turbulence (12), social contact (6), communication (6) and responsibility (5). The scale assesses broad and relevant constructs. Both professionals and non-professionals can administer the scale without special training and it can also be used by service providers. Each item is scored on a 4-point ordinal rating. The anchor points added to each item are: 1: no difficulty, 2: slight difficulty, 3: moderate difficulty and 4: extreme difficulty. A higher score means greater disability and malfunctioning. The test–re-test reliability was 0.89 for the total scale and 0.78–0.90 for the subscales.^20^ In addition, a subscale with six items on family harmony (Box 1) was included, raising the total number of items to 45. Scoring of these items was similar to that of other items of the scale. The test–re-test reliability of the subscale on family harmony was found to be 0.89.

**Box 1:** Six items in the subscale on family harmony

Disturbs family conversation Insists that all others in the family obey him/her Hostility targeted to a particular family member Does not show interest in events at relatives' homes Purposive behaviour to disturb key family carers Tries or threatens to run away from home

The original scale was independently translated into Malayalam by a clinical psychologist, three psychiatric social workers, one psychiatrist, and a language expert. All the six experts together corrected the minor discrepancies that occurred in translation. The corrected version was back translated to English by another group of five experts, proficient in both Malayalam and English. There were no major differences between the original and the translated versions. The same professional team translated and back-translated the subscale items on family harmony. The final version was administered to the subjects.

#### Patient Rejection Scale

Kreisman *et al*.^18^ developed an 11-item scale that assesses the rejection response of key family members of mentally ill patients. There are 5 positive and 6 negative items. The response categories for each item are ‘often’, ‘sometimes’ and ‘never’, and are scored as 1, 2 and 3, respectively. Reverse scoring is done for negative items. A higher score indicates greater rejection. The coefficient alpha is 0.78 for the English version,^18^ 0.93 for the translated Malayalam version^21^ and 0.72 for the German version. The cross-cultural validity of the scale is established.^22^

### Administration

The scales were administered to the key family carers when they accompanied their patient relative for follow up at the outpatient department of the Mental Health Centre, Thiruvananthapuram. To the key family carers of those attending the day-care centre, the scales were administered when they came to attend the weekly family group meetings at the centre. It took about 20–25 minutes for the administration of LSP and about 10–15 minutes for PRS.

## RESULTS

The LSP of the sample did not show gender differences in any of the subscale scores, the original total scale (OTS) score and the grand total scale (GTS) score, which included family harmony (Table 1). However, the mean OTS score of patients from urban areas was higher than those from rural areas. Significant differences were not seen between the LSP of patients attending and not attending the day-care centre. No association was found between LSP and income level.

**Table 1:** Life Skills Profile (LSP) scores and gender, locality and attendance of day-care centre

	Gender					Locality					Day-care centre				
			
	Male (*n*=29)		Female (*n*=19)			Urban (*n*=29)		Rural (*n*=19)			Attending		Not attending		
									
Subscales of LSP	Mean	SD	Mean	SD	t	Mean	SD	Mean	SD	t	Mean	SD	Mean	SD	t
Self-care	22.28	7.36	21.26	5.95	0.50	23.04	6.34	20.10	7.23	1.48	21.15	6.74	22.39	6.90	0.62
Non-turbulence	24.52	6.29	23.21	4.78	0.77	25.21	5.74	22.16	5.32	1.85	23.00	5.15	24.71	6.09	1.02
Social contact	17.41	3.31	17.00	2.56	0.48	17.69	2.41	16.58	3.49	1.31	17.40	3.25	17.14	2.68	0.30
Communication	13.24	5.04	11.47	4.03	1.28	13.10	4.92	11.68	4.35	1.02	11.85	4.20	13.04	5.05	0.86
Responsibility	9.97	3.12	9.47	2.89	0.56	10.69	2.77	8.37	2.79	2.83*	9.25	2.63	10.14	3.19	1.03
Family harmony	14.28	3.43	13.32	1.89	1.11	14.21	2.38	13.42	3.64	0.91	13.60	3.70	14.11	2.28	0.59
Grand total scale (GTS)	101.69	23.04	95.74	16.53	0.97	103.93	18.48	92.32	22.46	1.95	96.25	9.83	101.54	21.42	0.87
Original total scale (OTS)	87.41	20.54	82.42	15.53	0.90	89.72	16.80	78.90	19.98	2.03*	82.65	17.59	87.43	19.52	0.87

* significant at the 0.01 level

There was a significant correlation between LSP and PRS both for the OTS score (Pearson r: 0.37) and the GTS score (Pearson r: 0.39), which included the new subscale of family harmony; the scores were significant at the 0.01 level (Table 2).

**Table 2:** Correlation between Patient Rejection Scale and Life Skills Profile (LSP) (*n*=48)

Subscales of LSP	Pearson r
Self-care	0.30
Non-turbulence	0.32
Social contact	0.23
Communication	0.34*
Responsibility	0.30
Family harmony	0.37*
Grand total scale (GTS)	0.39*
Original total scale (OTS)	0.37*

* significant at the 0.01 level

Among the subscales, the scores on communication and family harmony were found to be positively related to the rejection response of key family carers. The rejection response of key family carers, its relation to sociodemographic variables and its cross-cultural comparison on a larger sample was reported in an earlier study.^21^

## DISCUSSION

The LSP of the present sample did not show any gender difference, which is similar to earlier findings.^7^ However, Winefield and Harvey^23^ reported that women with schizophrenia have poorer communication skills than men. But they too did not find any significant difference in other subscales. While Rosen *et al*.^7^ found a linear trend in the effect of age on the subscales of non-turbulence and responsibility, the present study did not show any such relationship. Patients from urban areas had low LSP when compared to those from rural areas. The score on the subscale of responsibility differentiated the urban and rural groups. This probably means that key family carers from urban areas expect patients with schizophrenia to be more independent compared to those from rural areas. The role expectations of patients from urban areas are more pronounced and that could be one of the reasons for this difference. However, this difference is dissipated when the new dimension of family harmony is also taken into consideration. Probably the patient equally affects key family carers living in rural areas with schizophrenia's functioning within the family. Ali and Bhatti^5^ did not find urban and rural families to be different in experiencing the burden.

The LSP of patients attending and not attending the day-care centre did not show any significant difference. The observation that patients attending the day-care centre do not have a better LSP may point to the quality of activities at the centre. However, it may be premature to conclude that the day-care centre has not been beneficial to patients without evaluating the variables of duration of attendance and the quality of service provided at the centre. Gopinath and Rao^16^ recommended that psychosocial management should be included in day-care centres to compensate for the disability and improve social functioning.

The significant correlation between a feeling of rejection and LSP, especially with communication and family harmony, has several implications. In our sociocultural context, individuals are bound with the family and living with the family is given great importance. Chakrabarthi *et al*.^3^ compared the burden on families of patients with affective and schizophrenic disorders, and found that disruption of family leisure was significantly greater in the group of patients with schizophrenia than the former group. Moreover, Srinivasan and Thara^24^ observed that the majority of patients with schizophrenia live with their families. Any event that could affect the harmony within the family is likely to elicit strong emotional reactions from other family members. This could be one of the reasons for the perception of burden and the subsequent feeling of rejection. Though the key family carers were more tolerant and accommodative of the patient's level of self-care, non-turbulence, social contact and responsibility, their feeling of rejection was related to communication and the patient's skill in maintaining family harmony. However, whether this feeling of rejection gets translated into rejection behaviour in real life requires further study. Another reason for the positive correlation between the feeling of rejection and LSP could be the lack of availability of adequate inpatient facilities for patients with active psychotic symptoms at the village level. As a result, the responsibility of attending to the acute symptoms of the patient with schizophrenia till professional help can be sought rests with the family,^24^ which the family might find stressful.

The care-giving dimensions perceived by the patient also assume importance. Involvement Evaluation Questionnaire (IEQ)^25^ is a tool to assess care-giving consequences. The reliability of five versions of IEQ was established^26^ and its intercultural validity was described in the domains of interpersonal tension, worry, urging and persuasion across five European countries.^27^ However, cultural bias in these countries in care-giving is a possible factor and van Wijngaarden *et al*.^27^ suggested the need for developing national norm groups. LSP may need to be correlated with the care-giving dimension of the relatives, so that it may be established that better care-giving leads to better LSP.

The stigma of mental illness is yet another factor which is likely to lead to the feeling of rejection among key family carers. Though professionals try to dispel misconceptions about mental illness, the cultural belief that it is caused by the possession of ‘evil spirit’ or ‘a wrath’ is deep-rooted; hence, it is rather difficult to change this attitude.^28^ Probably, efforts to disseminate information, similar to the attempts made by Penn *et al*.^29^ should be initiated. Gerace *et al*.^30^ described the siblings of a patient with schizophrenia experiencing a pervasive and dampening influence on their lives and in the lives of everyone in the family. Siblings have expressed a feeling of detachment from the patient, guilt about wanting to get away, yet impelled to do so for their own well-being. Winefield and Harvey^31^ suggested that the members of the family also need attention, information and support since caring for a family member with schizophrenia is a stressful task, frequently with little reward in terms of gratitude and social recognition.

Qureshi *et al*.^32^ observed that patients with schizophrenia hailing from joint families have a favourable outcome and attributed it to the emotional support and emotional buffer provided by the joint family. But in Kerala, as a result of various social changes, family structures are changing and joint families are becoming rare. Families are finding it difficult to care for the seriously mentally ill in the community.^33^ Therefore, psychosocial management has to focus on improving the life-skills functioning of patients and the emotional reaction of key family carers. Fadden^34^ developed behavioural family therapy (BFT) approaches for the treatment of schizophrenia, and several mental health personnel are trained in the system in the UK. In the Mediterranean setting too, family interventions have been found to have positive implications on relapse and social functioning. On evaluation, the BFT approach was found to have an advantage over intervention through family members' group.^35^ Though we talk a great deal about family traditions and systems in India, we are yet to develop a model of a family intervention programme for families of patients with schizophrenia which can be used by community mental health professionals. Developing a family intervention model that can be applied to culturally and linguistically diverse populations may help improve the LSP, reduce the feelings of rejection and being burdened, and ease the rehabilitation process.

One of the limitations of the study is that the life-skills measures were not correlated with the symptom profile of the patients. Correlating the scores of LSP with the measures of Scale for Assessment of Negative Symptoms (SANS), Scale for Assessment of Positive Symptoms (SAPS) or Positive and Negative Syndrome Scale (PANSS) would have yielded more meaningful data since negative symptoms in schizophrenia are likely to influence the LSP.^12^ Future studies may be conducted taking this into account so that it could help rehabilitate people with schizophrenia.
